# Influence of Receptor Polymorphisms on the Response to α-Adrenergic Receptor Blockers in Pheochromocytoma Patients

**DOI:** 10.3390/biomedicines10040896

**Published:** 2022-04-13

**Authors:** Annika M. A. Berends, Mathieu S. Bolhuis, Ilja M. Nolte, Edward Buitenwerf, Thera P. Links, Henri J. L. M. Timmers, Richard A. Feelders, Elisabeth M. W. Eekhoff, Eleonora P. M. Corssmit, Peter H. Bisschop, Harm R. Haak, Ron H. N. van Schaik, Samira el Bouazzaoui, Bob Wilffert, Michiel N. Kerstens

**Affiliations:** 1 Department of Endocrinology, University of Groningen, University Medical Center Groningen, Hanzeplein 1, 9713 GZ Groningen, The Netherlands; e.buitenwerf@umcg.nl (E.B.); t.p.links@umcg.nl (T.P.L.); m.n.kerstens@umcg.nl (M.N.K.); 2 Department of Clinical Pharmacy and Pharmacology, University of Groningen, University Medical Center Groningen, 9713 GZ Groningen, The Netherlands; m.s.bolhuis@umcg.nl (M.S.B.); y.g.m.mettes@rug.nl (B.W.); 3 Department of Epidemiology, University of Groningen, University Medical Center Groningen, 9713 GZ Groningen, The Netherlands; i.m.nolte@umcg.nl; 4 Department of Internal Medicine, Radboud University Medical Center, 6525 GA Nijmegen, The Netherlands; henri.timmers@radboudumc.nl; 5 Department of Endocrinology, Erasmus University Medical Center, 3015 GD Rotterdam, The Netherlands; r.feelders@erasmusmc.nl; 6 Department of Internal Medicine Section Endocrinology, Amsterdam University Medical Centers Location VUmc, 1117 HV Amsterdam, The Netherlands; emw.eekhoff@amsterdamumc.nl; 7 Department of Internal Medicine, Division of Endocrinology, Leiden University Medical Center, 2333 ZA Leiden, The Netherlands; e.p.m.van_der_kleij-corssmit@lumc.nl; 8 Department of Internal Medicine Section Endocrinology, Amsterdam University Medical Centers Location AMC, University of Amsterdam, 1105 AZ Amsterdam, The Netherlands; p.h.bisschop@amsterdamumc.nl; 9 Department of Internal Medicine, Máxima Medical Center, 5504 DB Eindhoven/Veldhoven, The Netherlands; h.haak@mmc.nl; 10 CAPHRI School for Public Health and Primary Care, Ageing and Long-Term Care, Maastricht University, 6229 HX Maastricht, The Netherlands; 11 Department of Internal Medicine, Division of General Internal Medicine, Maastricht University Medical Centre+, 6229 HX Maastricht, The Netherlands; 12 Department of Clinical Chemistry, Erasmus University Medical Center, 3015 GD Rotterdam, The Netherlands; r.vanschaik@erasmusmc.nl (R.H.N.v.S.); s.elbouazzaoui@erasmusmc.nl (S.e.B.); 13 Department of Pharmacotherapy, Epidemiology & Economics, Groningen Research Institute of Pharmacy, University of Groningen, 9713 GZ Groningen, The Netherlands

**Keywords:** pheochromocytoma, paraganglioma, single nucleotide polymorphism, adrenergic receptor, alpha-adrenergic receptor blocker, pharmacogenetics, personalized medicine

## Abstract

**Background:** Presurgical treatment with an α-adrenergic receptor blocker is recommended to antagonize the catecholamine-induced α-adrenergic receptor mediated vasoconstriction in patients with pheochromocytoma or sympathetic paraganglioma (PPGL). There is, however, a considerable interindividual variation in the dose-response relationship regarding the magnitude of blood pressure reduction or the occurrence of side effects. We hypothesized that genetically determined differences in α-adrenergic receptor activity contribute to this variability in dose-response relationship. **Methods:** Thirty-one single-nucleotide polymorphisms (SNPs) of the α1A, α1B, α1D adrenoreceptor (*ADRA1A*, *ADRA1B*, *ADRA1D*) and α2A, α2B adrenoreceptor (*ADRA2A, ADRA2B*) genes were genotyped in a group of 116 participants of the PRESCRIPT study. Haplotypes were constructed after determining linkage disequilibrium blocks. **Results:** The *ADRA1B* SNP rs10515807 and the *ADRA2A* SNPs rs553668/rs521674 were associated with higher dosages of α-adrenergic receptor blocker (*p* < 0.05) and with a higher occurrence of side effects (rs10515807) (*p* = 0.005). Similar associations were found for haplotype block 6, which is predominantly defined by rs10515807. **Conclusions:** This study suggests that genetic variability of α-adrenergic receptor genes might be associated with the clinically observed variation in beneficial and adverse therapeutic drug responses to α-adrenergic receptor blockers. Further studies in larger cohorts are needed to confirm our observations.

## 1. Introduction

Pheochromocytomas and sympathetic paragangliomas (PPGL) are rare neuroendocrine tumors localized in adrenal medulla and extra-adrenal sympathetic paraganglia, respectively [1]. The production and secretion of excessive amounts of catecholamines are cardinal features of PPGL and responsible for the associated increased cardiovascular risk [2,3,4,5]. Surgical resection of a PPGL is the only option for a cure, but it is known to be a high-risk procedure due the uncontrolled release of catecholamines [6]. In order to minimize the hyperadrenergic hemodynamic effects and prevent cardiovascular complications, pretreatment with an α-adrenergic receptor blocker is usually recommended to antagonize the catecholamine-induced α-adrenergic receptor mediated vasoconstriction [7]. 

The magnitude of blood pressure reduction or the development of side effects in response to a certain dose of an α-adrenergic receptor blocker displays a considerable interindividual variability. Moreover, serious intra-operative hemodynamic instability might still occur despite presurgical treatment with high doses of an α-adrenergic receptor blocker [8]. Variables explaining these interindividual differences in dose-response relationship are largely unknown at the moment. 

It is conceivable that genetically determined differences in α-adrenergic receptor activity contribute to the observed variation in the dose–response relationship. α-Adrenergic receptors (α-ARs) are G protein-coupled receptors (GPCRs) and can be classified according to their pharmacological specificity as alpha 1 (α_1_-AR) or alpha 2 (α_2_-AR) adrenergic receptors. Each comprises three subtypes encoded by genes on different chromosomes, denoted as α_1a_-AR (*ADRA1A*; chromosome 8), α_1b_-AR (*ADRA1B*; chromosome 5), α_1d_-AR (*ADRA1D*; chromosome 20), α_2a_-AR (*ADRA2A*; chromosome 10), α_2b_-AR (*ADRA2B*; chromosome 2), and α_2c_-AR (*ADRA2C*; chromosome 4). These subtypes are expressed in a wide range of tissues, including the central nervous system (predominantly ADRA1C, ADRA2A, ADRA2C), blood vessels (predominantly ADRA1, ADRA2B), and the heart (predominantly ADRA1C) [9,10,11,12,13,14]. The α-ARs in blood vessels play an important role in blood pressure regulation, as their activation results in vasoconstriction with increase of the peripheral vascular resistance [13,15,16]. Besides tissue-specific differences in distribution and expression levels of AR subtypes, naturally-occurring human single-nucleotide polymorphisms (SNPs) of the α-ARs can also contribute to the variability in α-AR-mediated physiological responses [17,18]. For instance, certain α-AR genes and polymorphisms have been associated with high blood pressure and increased cardiovascular risk [19,20,21,22]. The influence of genetic variants of the α_1_-AR or α_2_-AR on the response to an α-adrenergic receptor blocker or hemodynamic parameters, however, is largely unknown (Supplementary Table S1) [23].

We hypothesized that the response to the α-adrenergic receptor blockers in patients with PPGL is modulated by certain SNPs of the α-ARs gene. To this end, we evaluated in patients scheduled for PPGL resection the relationship between polymorphisms of the α-AR and the degree of perioperative hemodynamic control as well as the occurrence of side effects. 

## 2. Materials and Methods

### 2.1. Study Population and Design 

Study subjects participated in the PRESCRIPT study, a randomized controlled trial comparing presurgical treatment with either phenoxybenzamine, a nonselective and non-competitive α1- and α2- adrenergic receptor blocker, or doxazosin, a selective and competitive α1-adrenergic receptor blocker, in patients with PPGL (ClinicalTrials, number NCT01379898). The study was approved by the institutional review board of the University Medical Center Groningen, University Groningen, The Netherlands, in compliance with the Dutch Medical Research Involving Human Subjects Act and the Declaration of Helsinki. Written informed consent was provided by all participants. This study has been described in detail elsewhere [8]. In brief, the study population consisted of patients aged 18 years or older with non-metastatic PPGL. Past medical history of cardiovascular disease was recorded. All patients were randomized to either pretreatment with phenoxybenzamine or doxazosin. Pretreatment was started 2–3 weeks before surgery using blood pressure guided dose titration (Supplementary Table S2). Target values were blood pressure <130/80 mmHg in the supine position and a systolic blood pressure between 90–110 mmHg in the upright position. A calcium channel blocker was added when these targets were not reached despite maximum dosage of the α-adrenergic receptor blocker. A β-adrenergic receptor blocker was added in the case of heart rates >80 bpm or >100 bpm in the supine and upright position, respectively. In addition, a high-salt diet was advised and an infusion of 0.9% saline was administered within 24 h prior to surgery. Resection was postponed if the supine blood pressure was >160/100 mmHg on the day before surgery. The majority of patients were operated by minimal invasive surgical techniques (Table 1). Hemodynamic management during and after surgery was performed using a standardized operating procedure. Blood pressure and heart rate during surgery were monitored by continuous intra-arterial measurement. Intraoperative hemodynamic targets were systolic blood pressure <160 mmHg, mean arterial pressure (MAP) >60 mmHg, and heart rate <100 bpm. After surgery, patients were monitored at the post-anesthesia or intensive care unit.

### 2.2. Data Recording and Analysis

All data on blood pressure, heart rate, and medication was extracted from the electronic patient data monitoring system starting at first visit and ending at discharge from the post-anesthesia care unit or intensive care unit. Treatment follow-up was performed using a strict and standardized pretreatment protocol. During the whole pretreatment period, blood pressure and heart rate were measured twice daily with a certified automated electronic blood pressure monitor just before ingestion of the study drugs. Each measurement consisted of a single recording after 5 min of supine rest and subsequently after 3 min in upright posture. Side-effects of α-adrenergic receptor blockers were self-recorded by using a structured patient diary. Furthermore, both duration and amplitude of hemodynamic variables outside the target range were assessed, and cumulative dosage of vasoactive medication was calculated. The degree of intraoperative hemodynamic instability was assessed by using the hemodynamic instability score [24], which consists of three components: hemodynamic variables (i.e., blood pressure and heart rate), cumulative dosage of vasoactive medication, and volume therapy. A higher hemodynamic instability score represents a higher degree of overall hemodynamic instability.

### 2.3. DNA Collection and Genetic Analyses 

DNA was extracted and samples were diluted with a Tris-EDTA (TE) buffer to a volume of 50 µL with a minimum concentration of 10 ng/m. Samples were stored in a half-deep well plate (Thermo Scientific, Waltham, MA, USA, 0.8 mL 96 well storage plate, art.nr. AB-0765) protected with a removable heat seal and kept at −80 °C until analysis. 

All DNA samples were analyzed at the Department of Clinical Chemistry at the Erasmus Medical Center (Rotterdam, the Netherlands). All known single nucleotide polymorphisms (SNPs) of α-AR 1A (*ADRA1A*), 1B (*ADRA1B*), 1D (*ADRA1D*), 2A (*ADRA2A*), 2B (*ADRA2B*), and 2C (*ADRA2C*) were selected for analysis, resulting in a final list of 31 SNPs (Supplementary Table S1). For rs1048101, rs1383914, rs13278849 (*ADRA1A*), rs1800544, and rs1800545 (*ADRA2A*), genotyping was performed on the Life Technologies Taqman^®^ 7500 system (Applied Biosystems, Life Technologies Europe BV, Bleiswijk, The Netherlands). For the other 26 SNPs (see Supplementary Table S1), the Quantstudio 12K Flex (Thermo Fisher) was used. With this method, two probes, one for the wildtype and one for the variant sequence, are coupled with FAM or VIC reporter dyes, of which the fluorescent signal is measured at, respectively, 530 nm and 554 nm to distinguish between wild-type, heterozygote, or homozygote. Genotyping was carried out according to the manufacturer’s instructions. 

### 2.4. Statistical Analyses 

Continuous variables are described by their mean and standard deviation, when they are normally distributed, or by median and interquartile range, if their distributions were skewed. For categorical variables, counts and frequencies are presented. 

Firstly, the four outcome variables—dose of α-adrenergic receptor blockers, total number of side effects, hemodynamic instability score, and the cumulative time outside the blood pressure target range during surgery—were analyzed univariably with the potential confounders age, sex, body mass index, systolic blood pressure at baseline in supine position, total number of antihypertensive comedications at baseline, tumor size, plasma levels of catecholamines, serum creatinine, and randomization arm of the trial (i.e., treatment with either doxazosin or phenoxybenzamine). The latter two outcomes were analyzed using linear regression, for which the cumulative time outside the blood pressure target range during surgery was square root transformed to render a normal distribution. The outcomes dose of α-adrenergic receptor blockers and total number of side effects were categorical variables, and therefore ordinal regression was used for their association analysis. The various incremental dosages of each α-adrenergic receptor blocker were arbitrarily transformed into three incremental dosage steps (i.e., low, consisting of doxazosin 0–8 mg or phenoxybenzamine 0–20 mg; moderate, consisting of doxasozin 12–28 mg or phenoxybenzamine 40–90 mg; and high, consisting of doxasozin 32–48 mg or phenoxybenzamine 100–140 mg) to meet with the assumption of proportional odds (Supplementary Table S2). Total number of side effects were categorized in 0, 1, 2, 3, or ≥4 side effects. Covariables with a *p*-value below 0.2 were considered as confounders and included in subsequent analyses.

Secondly, the SNPs were associated with the outcomes using an additive model, which is that the effect of the homozygotes was modeled as being double the effect of heterozygotes, while adjusting for confounders. SNPs were excluded from the analyses if the quality of the SNP was regarded insufficient, based on the following criteria: a call rate (i.e., number of samples with a non-missing genotype) <80%, a minor allele frequency < 5%, or a deviation of the Hardy-Weinberg equilibrium (*p*-value < 0.05/31). The call rate per sample was calculated to determine the quality of the samples. For the SNP analyses, none of the samples was excluded.

In addition, haplotype analyses were performed. Haplotype blocks were constructed using the confidence intervals method in Haploview [25,26]. Within each block, haplotypes were constructed using the haplo.em() function from the haplo.stats package [27]. Only samples with a call rate ≥0.5 were included in this analysis (*n* = 110). The most likely haplotype combination was assigned to each individual, provided that the haplotype probability was >0.7. Otherwise, it was set to missing. Next, for each haplotype that occurred at least 10 times in the dataset, an association analysis was carried out using an additive model adjusting for covariables.

Two sensitivity analyses were performed: one using only the samples with a call rate >50% and one using only the European samples, to test if the quality of the samples or the ethnicity of the samples influenced the results.

Because we tested 24 SNPs, a multiple testing correction for statistical significance was required. Because SNPs were not all independent, linkage disequilibrium was calculated. SNPs in at least moderate linkage disequilibrium (r^2^ > 0.5) were considered to be dependent. This yielded 14 independent tests, so the *p*-value threshold for statistical significance was 0.05/14 = 0.0036. All analyses were performed using R version 3.6 [28].

## 3. Results 

Of the 134 patients who had participated in the PRESCRIPT trial, samples of 16 patients were not retrievable from the biobank. In addition, samples of two patients contained too little DNA for genotyping. Thus, SNP analysis was performed in 116 patients with either a pheochromocytoma (94%) or a sympathetic paraganglioma (6%). Baseline characteristics are shown in Table 1. Mean age of the study population was 55 ± 15.1 years, and the majority (93%) were of European ancestry. Side-effects of α-adrenergic receptor blockers were recorded as dizziness (*n* = 64), dry mouth (*n* = 12), dry eyes (*n* = 3), nasal congestion (*n* = 30), fatigue (*n* = 30), headache (*n* = 20), palpitations (*n* = 16), abdominal distension (*n* = 23), obstipation (*n* = 5), dyspnea (*n* = 7), urinary incontinence (*n* = 4), or peripheral edema (*n* = 8). 

Age, female sex, body mass index, systolic blood pressure at baseline in supine position, and total number of antihypertensive comedications were all nominally significantly associated with the dose of α-adrenergic receptor blockers (Table 2). No significant effect on the dose was observed for tumor size, plasma levels of total catecholamines, serum creatinine, or randomization arm. Only body mass index was significantly associated with the number of side effects in the multivariable model. The randomization arm of the trial was significantly associated with the hemodynamic instability score in the multivariable model, while body mass index, baseline systolic blood pressure in supine position, and plasma levels of total catecholamines showed a suggestive association. Total plasma levels of catecholamines were the only variable demonstrating a significant association with the cumulative intraoperative time outside the blood pressure target range.

Quality control of the SNP genotyping showed that three SNPs had an insufficient call rate. For four SNPs, the minor allele frequency was below 5%. All SNPs were in Hardy–Weinberg equilibrium, resulting in 24 SNPs left for analysis. The SNP association analyses adjusted for confounders revealed three SNPs that were nominally significantly associated with dose of α-adrenergic receptor blockers (rs10515807 (*p* = 0.047), rs521674 (*p* = 0.014), and rs553668 (*p* = 0.024)) (Table 3). The G alleles of rs10515807 in the *ADRA1B* gene and rs553668 in the *ADRA2A* gene both caused a three times lower risk of being in a higher dosage step than allele A (odds ratio (OR) = 0.31 and 0.26, respectively), while the T allele of rs521674 in *ADRA2A* was associated with a three times higher risk than the A allele (OR = 3.30). The associations remained unchanged when low quality samples were excluded but became less significant when only European samples were analyzed (Supplementary Table S3). SNP rs10515807 was also nominally associated with the number of side effects in the multivariable model (*p* = 0.005), and this association did not change when low-quality or non-European samples were removed (Table 3; Supplementary Table S4). However, none of these significances survived the multiple testing correction. No SNP associations were observed for the hemodynamic instability score or the cumulative intraoperative time outside the blood pressure target range in the cohort as a whole (Table 3).

**Figure 1:**
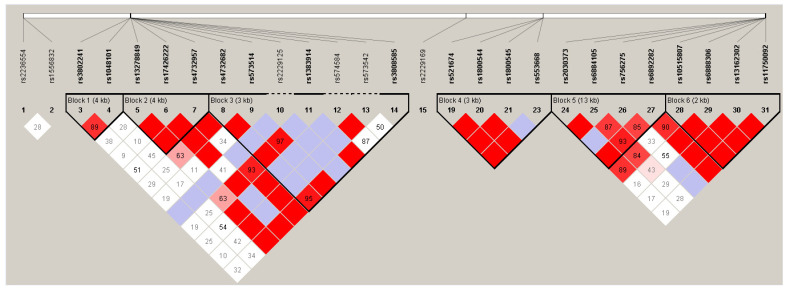
**Haplotype blocks within the candidate genes**. Linkage disequilibrium plot of the SNPs that were genotyped in *ADRA2D* (SNPs 1–2), *ADRA1A* (SNPs 3–14), *ADRA2B* (SNP 15), *ADRA2A* (SNPs 19–23), and *ADRA1B* (SNPs 24–31). The color scheme is a reflection of D′ (white meaning no linkage disequilibrium (D′ = 0) and red complete linkage disequilibrium (D′ = 1)). Haplotype blocks have been calculated using the confidence intervals method (Gabriel 2002). Numbers inside the squares refer to values of D′, with no number indicating complete linkage disequilibrium. SNP = single nucleotide polymorphism.

Linkage disequilibrium analyses showed that, within the *ADRA1A* gene, three haplotype blocks could be determined: one block within the *ADRA2A* gene and two blocks within the *ADRA1B* gene (Figure 1). The haplotype analyses revealed nominally significant associations of haplotype A-C-A-C in block 6, consisting of SNPs rs10515807, rs6888306, rs13162302, and rs11750092 in the *ADRA1B* gene with both a higher dose of α-adrenergic receptor blockers (OR = 3.30; *p* = 0.044) and a higher number of side effects (OR = 3.51; *p* = 0.007) (Table 4). Another haplotype in the same block (G-C-A-C), that differs only in the first position (i.e., rs10515807), was associated with a lower number of side effects (OR = 0.55; *p* = 0.049) (Table 4). These associations did, however, not survive multiple testing correction. No haplotype associations were observed with the hemodynamic instability score and the cumulative intraoperative time outside the blood pressure target range (Table 4). 

## 4. Discussion

In this study, we investigated, in a well-defined group of patients undergoing resection of a PPGL, whether polymorphisms of the α-ARs genes affect the clinical response to presurgical administration of α-adrenergic receptor blockers. Our findings showed that patients carrying minor alleles for a SNP in the intron region (rs10515807-A) of the *ADRA1B* gene or for SNPs in the three prime untranslated region (rs553668-A) or the 2kb upstream region (rs521674-T) of the *ADRA2A* gene needed a higher dosage of an α-adrenergic receptor blocker. In addition, it was found that patients with the A allele of the rs10515807 SNP seemed to be more prone to developing α-adrenergic receptor blocker-related side-effects, independently of the prescribed dosage. Haplotype analysis produced additional evidence for this relationship, with predominantly a role for the *ADRA1B* gene. However, none of these associations remained significant after correction for multiple testing. 

AR genes are highly polymorphic and demonstrate genetic variations in both coding and non-coding regions. Adrenoreceptors are the target for several frequently prescribed drugs, especially in cardiovascular medicine, and represent pharmacodynamic candidate genes [12]. To date, only a few small-sized studies have addressed the potential clinical consequences of polymorphisms of the genes encoding adrenergic receptors [29]. Most available studies were focused on beta adrenergic receptors (β-ARs) and to a lesser extent on α_2_–AR (*ADRA2A*, *ADRA2B*, *ADRA2C*) (Supplementary Table S1) [14,16,17,30]. 

The human *ADRA1B* gene consists of two exons separated by a single large intron of 20 kb that interrupts the coding region at the end of the putative sixth transmembrane domain [31]. Thus far, data on the potential relationship between polymorphisms of the *ADRA1B* gene and the efficacy of α-adrenergic receptor blockers are very limited. It has been shown that prazosin, an α1-adrenergic receptor blocker, binds with equal affinity to both *ADRA1B* and *ADRA1A*, the latter being the principal mediator of vasoconstriction [13,32]. In a study among normotensive and hypertensive subjects, no relationship was found between four exonic *ADRA1B* polymorphisms and the blood pressure response to intravenous administration of the ADRA1B agonist phenylephrine [31]. In contrast, an intronic variant (rs10070745) of *ADRA1B* present in African Americans was associated with an enhanced vasoconstrictor response to phenylephrine [33]. The present study is the first to suggest a decreased efficacy of α-adrenergic receptor blockers as well as an increased susceptibility to adverse effects to these antihypertensive agents in carriers of the intronic G > A variant in rs10515807. It could be postulated that this polymorphism results in a decreased affinity of the ADRA1B, which would explain the need of a higher drug dose. Such a change in receptor affinity, however, would not provide an explanation for the observed association between this polymorphism and the enhanced susceptibility to adverse effects, which was also independent of the dose. Possible explanations could include, e.g., modulation of crosstalk between certain SNPs or cosegregation with other SNPs affecting pathways involved in the development of adverse effects, but these suggestions remain quite speculative. Additional studies are needed to further elucidate the functional consequences of these SNPs. 

The human *ADRA2A* gene is intronless and consists of one single 3650-base pair (bp) exon, which contains a 1353-bp open reading frame encoding a receptor protein of 450 amino acid residues [34]. Activation of the presynaptic ADRA2A results in a decrease of blood pressure and heart rate through negative feedback inhibition of the catecholamine secretion. *ADRA2A* knock-out mice were found to demonstrate a hyperadrenergic phenotype with elevated blood pressure and diminished hypotensive response to administration of clonidine [35]. We found that two *ADRA2A* SNPs, i.e., rs553668, formerly described as the *DraI* restriction fragment length polymorphism (RFLP), and rs521674, were associated with a higher requirement of α-adrenergic receptor blockers, suggesting that these polymorphisms result in a decreased inhibition of the presynaptic catecholamine release. This is more or less in agreement with a previous study demonstrating that carriers of the variant allele of rs553668 experienced a less pronounced blood pressure drop during exercise [36]. Of interest, in vitro experiments with human neuronal cells demonstrated that transfection with the rs553668 variant was associated with a decreased protein expression in subjects from European ancestry [37]. Thus, the higher requirement of α-adrenergic receptor blockers in patients with pheochromocytoma harboring the rs553668 polymorphisms of the *ADRA2A* gene could be due to a lower presynaptic receptor density. The relationship between blood pressure or antihypertensive drug response and the rs521674 polymorphism of the *ADRA2A* gene has not been described before and requires further investigations for determining the possible underlying mechanism. 

We were unable to find an association between α-AR variants and the hemodynamic profile during surgical resection of the PPGL. This might be explained by the fact that the primary endpoint of the PRESCRIPT study, defined as the cumulative intraoperative time of blood pressure outside the target range, also did not reach significance [8]. Intraoperative blood pressure during PPGL resection is affected by many different factors, including general health status, catecholamine secretion, and vaso-active drugs administration. Consequently, to identify the influence of a genetic polymorphism amidst these complex and interacting factors would require a substantial effect size of such a variant in order to be demonstrated. 

Our study had several strengths and limitations. A major strength of the current study is that we used a comprehensive prospective data collection derived from the only randomized controlled trial examining the efficacy of α-adrenergic receptor blockers in a large group of patients with a PPGL. In addition, this is the first study evaluating the relationship between the therapeutic response of α-adrenergic receptor blockers in patients who underwent a PPGL resection. Moreover, we used haplotype analysis, which can identify susceptibility loci that are not captured by single genetic variation test alone [25,38]. 

There are, however, also limitations that need to be addressed. As indicated earlier, we found nominally significant associations for three variants, but none of these associations remained significant after correction for multiple testing. This could be due to a lack of statistical power, despite the fact that the study population is one of the largest of its kind. As a result, our findings should be mainly considered as hypothesis generating and require validation in larger clinical cohorts. We did not investigate SNPs of the ADRA2C gene, but most study participants were white subjects, and polymorphisms of this gene are infrequent in a white population [12,29]. Moreover, we focused on SNPs concerning genes of the receptor itself, assuming these are the major contributors. One disadvantage of such an approach is that the complex system of the biology of drug actions in vivo probably may not be fully addressed. Additionally, there could be physiological relevant signaling pathways for this α-AR subtypes that have not been elucidated yet, and polymorphisms in genes contributing to the signal transduction of these GPCRs could also be of interest. 

In conclusion, this study indicates that genetic variants in *ADRA1B* and *ADRA2A* could modify α-adrenergic receptor blocker efficacy and the risk of developing side effects in PPGL patients pretreated with α-adrenergic receptor blockers. Future studies in larger cohorts are required to confirm our observations, which could open the way to personalized medicine based on pharmacogenetics in the management of patients with a PPGL. 

## Figures and Tables

**Table 1:** Baseline characteristics of the study population.

	All Subjects (*n* = 116)
***Demographics***	
Male sex—number (%)	51 (44)
Ethnicity	
European (%)	108 (93)
Asian (%)	3 (3)
African (%)	2 (1.5)
Latin American (%)	2 (1.5)
Arab (%)	1 (1)
Age (years)	55 ± 15.1
BMI (kg/m^2^)	25.9 ± 4.8
Serum creatinine (μmol/L)	76.1 ± 21.7
***Tumor characteristics***	
Pheochromocytoma—number (%)	109 (94.0)
sPGL—number (%)	7 (6.0)
Germline mutations—number (%)	23 (19.8)
Tumor size (mm)	53.63 (17.50–160.00)
Total plasma catecholamines (*n* < 5.28 nmol/L)	6.01 (3.53–17.26)
***Surgical approach***	
Laparoscopy—number (%)	82 (70.7)
Laparotomy—number (%)	20 (17.2)
Posterior retroperitoneoscopic—number (%)	14 (12.1)
***Pretreatment***	
Doxazosin/Phenoxybenzamine—number (%)	59 (51)/57 (49)
4/10 mg	3 (2.6)
8/20 mg	5 (4.3)
12/40 mg	5 (4.3)
16/60 mg	9 (7.8)
20/70 mg	2 (1.7)
24/80 mg	6 (5.2)
28/90 mg	1 (0.9)
32/100 mg	19 (16.4)
36/110 mg	2 (1.7)
40/120 mg	14 (12.1)
48/140 mg	50 (43.1)
Total number of side effects	2.0 (1.0–3.0)
***Presurgical hemodynamics***	
Supine SBP preoperative (mmHg) *	127.7 ± 19.1
Upright SBP preoperative (mmHg) *	118.2 ± 19.3
Heart rate baseline (bpm)	73.0 ± 12.0
***Intraoperative hemodynamics***	
Hemodynamic instability score	43.5 (30.3–59.0)
Time outside BP range (%)	10.0 (4.3–19.8)

Data are presented as number of patients (%), as mean with standard deviation, or as median with interquartile range. * With α-adrenergic receptor blockade. Abbreviations: SBP, systolic blood pressure; DBP, diastolic blood pressure; sPGL, sympathetic paraganglioma; bpm, beats per minute; BMI, body mass index.

**Table 2:** Associations of covariates with dose of α-adrenergic receptor blockers, number of side effects, hemodynamic instability score, and cumulative intraoperative time outside the blood pressure target range.

Outcome	Covariate	Beta	SE	Univariate *p*-Value	Multivariate *p*-Value
Dose of α-adrenergic receptor blockers	Age	0.046	0.013	*0.00092*	0.094
	Sex (female)	−0.77	0.38	*0.044*	*0.025*
	BMI	0.19	0.053	*0.00059*	*0.011*
	SBP baseline (supine)	0.047	0.0106	*0.000029*	*0.0013*
	Number of antihypertensive comedication day -1 (baseline)	0.92	0.28	*0.0014*	0.069
	Serum creatinine	0.0057	0.0086	0.50	n.a.
	Tumor size	−0.00024	0.00063	0.70	n.a.
	Catecholamines	0.0087	0.0109	0.43	n.a.
	Randomization	−0.089	0.36	0.80	n.a.
Number of side effects	Age	−0.003	0.011	0.78	n.a.
	Sex (female)	0.33	0.33	0.32	n.a.
	BMI	−0.066	0.034	0.06	*0.045*
	SBP baseline (supine)	0.004	0.007	0.55	n.a.
	Number of antihypertensive comedication day -1 (baseline)	0.39	0.22	0.07	0.16
	Serum creatinine	−0.003	0.008	0.74	n.a.
	Tumor size	−0.0003	0.0006	0.61	n.a.
	Catecholamines	0.012	0.009	0.17	0.38
	Randomization	−0.43	0.33	0.20	0.29
	Dose of α-adrenergic receptor blockers	0.15	0.24	0.53	n.a.
Hemodynamic instability score	Age	0.23	0.14	0.11	0.22
	Sex (female)	−1.39	4.32	0.75	n.a.
	BMI	−0.69	0.45	0.13	0.15
	SBP baseline (supine)	0.21	0.09	*0.018*	0.11
	Number of antihypertensive comedication day -1 (baseline)	1.76	2.70	0.52	n.a.
	Serum creatinine	−0.10	0.10	0.30	n.a.
	Tumor size	0.011	0.007	0.11	0.40
	Catecholamines	0.36	0.12	*0.0032*	0.15
	Randomization	9.63	4.20	*0.024*	*0.026*
Cumulative intraoperative time outside the blood pressure target range	Age	−0.013	0.62	0.25	n.a.
	Sex (female)	−0.28	0.33	0.40	n.a.
	BMI	−0.038	0.035	0.28	n.a.
	SBP baseline (supine)	0.003	0.007	0.67	n.a.
	Number of antihypertensive comedication day -1 (baseline)	0.23	0.21	0.28	n.a.
	Serum creatinine	−0.0027	0.0076	0.73	n.a.
	Tumor size	0.00054	0.00056	0.34	n.a.
	Catecholamines	0.02	0.0093	*0.031*	*0.031*
	Randomization	0.063	4.12	0.33	n.a.

SE, standard error; n.a., not applicable.

**Table 3:** Association of the SNPs with dose of α-adrenergic receptor blockers, number of side effects, the hemodynamic instability score, and the cumulative intraoperative time outside the blood pressure target range.

		Dose of α-Adrenergic Receptor Blockers		Number of Side Effects		Hemodynamic Instability Score		Cumulative Intraoperative Time Outside the Blood Pressure Target Range	
SNP-Allele	AF	OR (SE)	*p*-Value	OR (SE)	*p*-Value	Beta (SE)	*p*-Value	Beta (SE)	*p*-Value
rs2229169-T	0.328	0.73 (0.37)	0.39	0.98 (0.27)	0.95	0.36 (3.31)	0.91	−0.04 (0.26)	0.87
rs2030373-C	0.778	0.52 (0.46)	0.17	0.58 (0.37)	0.14	0.58 (4.17)	0.89	0.02 (0.33)	0.96
rs6884105-G	0.644	0.67 (0.40)	0.31	0.95 (0.28)	0.85	0.49 (3.29)	0.88	0.06 (0.26)	0.82
rs756275-T	0.073	0.70 (0.67)	0.59	1.01 (0.54)	0.98	3.18 (5.97)	0.60	−0.13 (0.46)	0.78
rs6892282-T	0.440	1.35 (0.35)	0.39	1.58 (0.27)	0.10	−3.53 (3.39)	0.30	−0.50 (0.25)	0.05
rs10515807-G	0.862	0.31 (0.58)	0.047 *	0.27 (0.46)	0.005 *	−2.18 (5.09)	0.67	0.26 (0.38)	0.50
rs6888306-T	0.248	0.89 (0.38)	0.77	1.23 (0.32)	0.52	−4.67 (3.77)	0.22	−0.28 (0.28)	0.32
rs13162302-G	0.196	1.03 (0.40)	0.94	1.27 (0.33)	0.47	−6.79 (4.02)	0.10	−0.43 (0.30)	0.15
rs11750092-T	0.192	1.20 (0.40)	0.66	1.36 (0.33)	0.36	−7.56 (4.02)	0.06	−0.46 (0.30)	0.15
rs3802241-G	0.545	1.28 (0.35)	0.48	1.19 (0.26)	0.51	−0.33 (3.31)	0.92	0.08 (0.23)	0.72
rs1048101-T	0.539	1.43 (0.31)	0.26	0.99 (0.24)	0.96	−2.16 (3.00)	0.47	−0.16 (0.23)	0.48
rs13278849-G	0.263	0.92 (0.34)	0.80	1.12 (0.28)	0.68	−0.08 (3.38)	0.98	−0.18 (0.27)	0.51
rs17426222-T	0.286	2.04 (0.45)	0.12	1.16 (0.32)	0.64	−1.72 (4.00)	0.67	0.18 (0.30)	0.56
rs4732957-C	0.784	1.50 (0.42)	0.34	1.06 (0.34)	0.87	1.17 (4.19)	0.78	0.31 (0.32)	0.35
rs4732682-T	0.458	0.78 (0.37)	0.50	0.95 (0.27)	0.86	1.27 (3.33)	0.71	0.03 (0.26)	0.91
rs573514-G	0.446	1.92 (0.37)	0.09	1.04 (0.28)	0.90	−2.77 (3.30)	0.40	0.16 (0.25)	0.52
rs1383914-T	0.530	1.41 (0.32)	0.29	1.21 (0.24)	0.43	−2.64 (3.00)	0.38	−0.02 (0.23)	0.95
rs3808585-T	0.250	0.74 (0.40)	0.46	1.31 (0.29)	0.36	2.34 (3.59)	0.52	−0.04 (0.29)	0.89
rs521674-T	0.260	3.30 (0.48)	0.014 *	1.04 (0.32)	0.91	1.41 (4.04)	0.73	−0.08 (0.31)	0.80
rs1800544-G	0.254	2.01 (0.38)	0.07	1.23 (0.29)	0.48	−0.11 (3.57)	0.98	−0.02 (0.28)	0.95
rs1800545-A	0.103	1.34 (0.56)	0.60	0.87 (0.43)	0.75	4.92 (5.43)	0.37	0.40 (0.41)	0.34
rs553668-G	0.859	0.26 (0.59)	0.024 *	0.72 (0.38)	0.39	1.12 (4.66)	0.81	0.27 (0.35)	0.44
rs2236554-T	0.643	0.85 (0.40)	0.69	1.11 (0.31)	0.73	−5.24 (4.06)	0.20	−0.34 (0.30)	0.27
rs1556832-T	0.505	0.63 (0.36)	0.20	0.87 (0.25)	0.58	−4.75 (3.15)	0.14	−0.18 (0.24)	0.46

AF, allele frequency; OR, odds ratio; SE, standard error, *, nominal significant.

**Table 4:** Haplotype analyses.

			Dose of α-Adrenergic Receptor Blockers		Number of Side Effects		Hemodynamic Instability Score		Cumulative Intraoperative Time Outside Blood Pressure Target Range	
Gene	Block ^#^	Haplotype	OR (SE)	*p*-Value	OR (SE)	*p*-Value	Bèta (SE)	*p*-Value	Bèta (SE)	*p*-Value
*ADRA1A*	1	A-C	0.70 (0.34)	0.30	0.93 (0.27)	0.78	0.79 (3.31)	0.81	−0.04 (0.25)	0.87
	1	G-T	1.58 (0.32)	0.15	1.12 (0.24)	0.63	−1.19 (2.94)	0.69	0.07 (0.23)	0.76
*ADRA1A*	2	A-C-C	0.62 (0.37)	0.20	0.85 (0.27)	0.56	1.49 (3.38)	0.66	−0.04 (0.26)	0.88
	2	A-T-C	1.95 (0.45)	0.14	1.13 (0.32)	0.71	−1.55 (4.06)	0.70	0.17 (0.30)	0.56
	2	G-C-A	0.81 (0.43)	0.63	1.31 (0.33)	0.42	−0.89 (3.91)	0.83	−0.22 (0.31)	0.48
*ADRA1A*	3	C-A-T-C	0.72 (0.58)	0.57	1.42 (0.51)	0.49	3.68 (5.64)	0.52	−0.54 (0.44)	0.22
	3	C-G-T-C	1.74 (0.36)	0.13	1.06 (0.28)	0.84	−2.46 (3.28)	0.46	0.18 (0.25)	0.48
	3	T-A-C-C	0.78 (0.37)	0.51	0.62 (0.31)	0.13	−1.95 (3.90)	0.62	−0.01 (0.29)	0.97
	3	T-A-C-T	0.87 (0.41)	0.74	1.18 (0.30)	0.58	2.81 (3.63)	0.44	0.00 (0.29)	0.99
*ADRA1B*	5	A-A-C-T	1.93 (0.46)	0.16	1.86 (0.37)	0.10	−1.45 (4.12)	0.73	−0.08 (0.33)	0.80
	5	C-A-T-T	1.58 (0.86)	0.60	0.58 (0.55)	0.33	6.49 (6.50)	0.32	−0.19 (0.49)	0.70
	5	C-G-C-G	0.71 (0.36)	0.34	0.66 (0.28)	0.15	3.65 (3.29)	0.27	0.35 (0.26)	0.19
	5	C-G-C-T	1.71 (0.71)	0.45	1.85 (0.50)	0.22	−5.56 (6.22)	0.37	−0.69 (0.46)	0.14
*ADRA1B*	6	A-C-A-C	3.30 (0.58)	0.044 *	3.51 (0.46)	0.007 *	2.54 (5.06)	0.62	−0.30 (0.38)	0.43
	6	G-C-A-C	0.72 (0.38)	0.38	0.55 (0.30)	0.05	3.04 (3.76)	0.42	0.38 (0.28)	0.18
	6	G-T-A-C	0.49 (0.85)	0.40	0.83 (0.67)	0.78	4.66 (7.84)	0.55	0.42 (0.56)	0.46
	6	G-T-G-T	1.02 (0.42)	0.96	1.16 (0.34)	0.67	−7.82 (4.17)	0.07	−0.38 (0.31)	0.23
*ADRA2A*	4	A-C-G-G	0.47 (0.39)	0.056	0.94 (0.30)	0.83	−0.36 (3.65)	0.92	0.11 (0.28)	0.69
	4	T-G-G-A	2.71 (0.51)	0.055	1.33 (0.37)	0.44	−3.60 (4.53)	0.43	−0.34 (0.35)	0.33
	4	T-G-A-G	1.55 (0.57)	0.44	0.81 (0.44)	0.62	5.79 (5.46)	0.29	0.19 (0.41)	0.65

^#^ Block 1, rs3802241-rs1048101; block 2, rs13278849-rs17426222-rs4732957; block 3, rs4732682-rs573514-rs1383914-rs3808585; block 4, rs521674-rs1800544-rs1800545-rs553668; block 5, rs2030373-rs6884105-rs756275-rs6892282; block 6, rs10515807-rs6888306-rs13162302-rs11750092. OR, odds ratio; SE, Standard error; * nominal-significant (*p* < 0.05).

## Data Availability

All data generated or analyzed during this study are included in this published article (and its Supplementary Materials).

## Supplementary Materials

The following are available online at https://www.mdpi.com/article/10.3390/biomedicines10040896/s1, Table S1: Overview of the single nucleotide polymorphisms of the alpha 1 and alpha 2 adrenergic receptor evaluated in the present study and the associated clinical conditions that have been reported in the literature, Table S2: Standardized incremental dosage steps for doxasozin and phenoxybenzamine, Table S3: Sensitivity analyses for dose of alpha adrenergic receptor blockers, Table S4: Sensitivity analyses for number of side effects. References [17,22,23,25,30,39,40,41,42,43,44,45,46,47,48,49,50,51,52,53,54,55,56,57,58,59,60,61,62,63,64,65,66,67,68,69,70,71,72,73,74,75,76,77,78,79,80,81,82,83,84,85,86,87,88,89] are cited in the Supplementary Materials.
