# Supplementary material for: Influence of Receptor Polymorphisms on the Response to α-Adrenergic Receptor Blockers in Pheochromocytoma Patients
Source: Biomedicines. 2022 Apr 13;10(4):896. doi: 10.3390/biomedicines10040896 (PMC9028965; doi:10.3390/biomedicines10040896)
Supplement: Supplementary file 1 [file biomedicines-10-00896-s001.zip › biomedicines-1661836-supplementary.pdf]

Supplementary Table S1: Overview of the single nucleotide polymorphisms of the α1- and α2-adrenergic receptor evaluated in the present study and the associated clinical conditions that have been reported in the literature

Receptor	SNP	Alleles	Gene consequence (variant)	Functional consequences (research model)	Clinical significance (in ClinVar)	Associated clinical conditions/ diseases*	Investigated associations with cardiovascular conditions	References
ADRA1A	rs1048101	T>C	Missense	No alteration of ligand binding / receptor activation found (human SNPs investigated in rat-1 fibroblast)	not reported	-autoimmune thyroid disease<br>-fibromyalgia<br>-CRPS I after distal radius fracture<br>-cocaine abuse, T-allele carriers ↑ reduction of cocaine use after treatment with disulfiram<br>-cervical vertigo	-associated with heart rate reactivity to stress (only in men, black adolescents and young adult population)<br>-associated (+) with max. SBP in men (CC) during exercise test<br>-associated (+) with major adverse cardiac events while on statins<br>- phenylephrine-mediated venoconstriction in local dorsal hand vein: no effect (European and African- American population)	[23,39–48]

							-exercise muscle vasodilation (forearm): no effect<br>- vasovagal syncope and positive HUTT test: no effect	
	rs13278849	G>A	Intron	unknown	not reported	-	unknown	
	rs1383914	T>C	non coding transcript	unknown	not reported	fibromyalgia	vasovagal syncope and positive HUTT test: no effect	[39,48]
	rs17426222	C>T	Intron	unknown	not reported	ADHD, generalized anxiety disorder	unknown	[49,50]
	rs3808585	C>T	Intron	unknown	not reported	ADHD	unknown	[49]
	rs4732682	C>T	Intron	unknown	not reported	ADHD	unknown	[49]
	rs4732957	A>C	Intron	unknown	not reported	-	Unknown	
	rs573514	A>G	Intron	unknown	not reported	ADHD, generalized anxiety disorder	unknown	[49,50]
	rs573542	C>T	Intron	unknown	not reported	-	vasovagal syncope and positive HUTT test: no effect	[50]
	rs574584	C>T	Intron	unknown	not reported	fibromyalgia	-no correlation with ADRA1 autoantibody production in patients with HT<br>-vasovagal syncope and positive HUTT test: no effect	[39,48,51]

	rs3802241	G>A	Intron	unknown	not reported	cervical vertigo	possible correlation with ADRA1 autoantibody production in patients with HT	[47,51]
	rs2229125	A>C	Missense	decreased antagonist (phentolamine) binding compared with WT, *K*I ↑ 3-fold (human SNPs investigated in rat-1 fibroblast)	not reported no major effects on receptor expression or stability	-	because of low frequencies (pilot population) direct association with hypertensive disease states not possible	[23]
ADRA1B	rs10515807	G>A	Intron	unknown	not reported	asthma (African-ancestry population)	unknown	[52]
	rs11750092	C>T	Intron	unknown	not reported	-	unknown	
	rs13162302	A>G	Intron	unknown	not reported	ADHD	unknown	[53]
	rs2030373	C>A	Intron	unknown	not reported	ADHD	unknown	[53]
	rs6884105	G>A	Intron	unknown	not reported	ADHD	unknown	[53]
	rs6888306	C>T	Intron	unknown	not reported	ADHD	unknown	[53]
	rs6892282	G>T	Intron	unknown	not reported	ADHD	unknown	[53]
	rs756275	C>T	Intron	unknown	not reported	ADHD	unknown	[53]
ADRA1D	rs1556832	C>T	Intron	unknown	not reported	IBS (GI symptom severity, ↑ brain regions linked to pain perception)	unknown	[54]

	rs2236554	T>A	3 prime UTR	unknown	not reported	-↑ clinical response of metoclopramide without more side-effects, in patients with gastroparesis<br>-T-allele carriers ↑ reduction of cocaine use after treatment with doxazosin	unknown	[55,56]
ADRA2A	rs1800544	G>C	2KB upstream	↓ transcription and receptor expression compared to WT (haplotype 3, investigated in BE(2)-C human neuroblastoma cells)	benign	-obesity<br>-clozapine-/olanzapine-induced weight gain (C-allele, European population)<br>-ADHD and MPH effects<br>-schizophrenia<br>-social withdrawal<br>-↑ insulin and HOMA-IR index (G-allele, Russian female Tartar population)<br>- delayed discounting in	-significantly smaller hypotensive responses to dexmedetomidine (selective ADRA2A agonist) (carriers of haplotype 3)<br>-vascular reactivity (↑ vasoconstriction) to cold and psychological stress (G allele carriers, young black population)	[30,42,57–71]

						cocaine users (G-allele)<br>-severity of breast cancer<br>-stress induced hyperglycemia		
	rs1800545	G>A	5 prime UTR	unknown	not reported	↑ risk of DVT (population with sticky platelet syndrome)	hypotensive response to dexmedetomidine (selective ADRA2A agonist): no effect	[25,72]
	rs553668	A>G	3 prime UTR	↑ transcription and receptor expression (haplotype 4, investigated in BE(2)-C human neuronal cells)	not reported	-↑ BMI and body fat (G allele, African-American population)<br>-exercise induced fat loss (Polish women)<br>-satiation and gastric motor function<br>-ADHD (+/-tics) and response time variability of CPT and treatment<br>-blood glucose DM2 risk, stress induced	-significantly ↑ hypotensive responses to dexmedetomidine (selective ADRA2A agonist) (homozygous carriers of minor allele, haplotype 4)<br>-associated with less ↓SBP and DBP during exercise when carrying the variant allele<br>-hypertension<br>-muscle vasodilation in men (forearm)	[30,57,59,60,66,73–83]

						hyper- glycemia and metabolic traits<br>-osteoporosis<br>-severity of breast cancer<br>-birth weight		
	rs11195419	C>A	3 prime UTR	unknown	not reported	-↑ suicidality among men during antidepressant treatment (nortriptyline)<br>-ADP-induced platelet aggregation in men with DAPT (↓ADP inhibition, Chinese population)	association (+) with SBP/DBP and HT, especially in individuals with BMI≥25 kg/m^2 and smokers	[22,84–85]
	rs521674	T>A	2KB upstream	unknown	not reported	alcoholism<br>steeper delayed discounting in cocaine users (T-allele)	no association with anti-hypertensive drug responses in essential HT (amlodipine, bisoprolol, HCT and losartan monotherapy)	[70,86,87]
ADRA2B	rs4066772	del/dup(CTC)$_2$TTC (del301-303 variant)	inframe indel	encodes a receptor that manifests loss of short-term agonist-induced	benign	-	↑vascular desensitization to dexmedetomidine (selective ADRA2A	[17,88–89]

				desensitization *in vitro* (investigated in CHO cells)			agonist) *in vivo* in case of ins/ins	
	rs786205528	G>A	stop gained	unknown	likely pathogenic	-	unknown	
	rs2229169	T>G	synonymous	unknown	not reported	-	↑vascular desensitization to dexmedetomidine (selective ADRA2A agonist) *in vivo* when homozygous for major allele (*high linkage disequilibrium with rs4066772)	[88]
	rs879255577	delAAAG(C)$_4$ACCA-insCTGCCAAAC	inframe indel	gain of function effect due to loss of spinophilin interaction (investigated in *Xenopus laevis* oocytes)	VUS	autosomal dominant cortical myoclonus and epilepsy	unknown	[89]

Alternative description for 3’ and 5’- UTR could be 3’ regulatory region and 5’ regulatory region

ClinVar: database of assertions about the clinical significance and phenotype relationship of human variation

*only associations that were statistically significant with certain diseases or conditions are reported

Abbreviations: CRPS I: complex regional pain syndrome type 1, HUTT: head-up tilt table, ADHD: attention-deficit hyperactivity disorder, HT: hypertension, WT: wild type, IBS: irritable bowel syndrome, GI: gastrointestinal, UTR: untranslated region, Indel: insertion and deletion, VUS: variance of unknown significance, DVT: deep vein thrombosis, SBP: systolic blood pressure, DBP: diastolic blood pressure, DAPT: Dual Antiplatelet Therapy, HCT: hydrochlorothiazide, CHO: Chinese hamster ovary, MPH: methylphenidate, CPT: continuous performance test, DM2: diabetes mellitus type 2.

References

1. Loyd, R.V.; Osamura, R.Y.; Kloppel, G.; Rosai, J. *WHO Classification of Tumours: Pathology and Genetics of Tumours of Endocrine Organ*, 4th ed; IARC, Lyon France, 2017.
2. Stolk, R.F.; Bakx, C.; Mulder, J.; Timmers, H.J.; Lenders, J.W. Is the excess cardiovascular morbidity in pheochromocytoma related to blood pressure or to catecholamines? *J. Clin. Endocrinol. Metab.* **2013**, *98*, 1100–1106.
3. Giavarini, A.; Chedid, A.; Bobrie, G.; Plouin, P.F.; Hagege, A.; Amar, L. Acute catecholamine cardiomyopathy in patients with pheaochromocytoma or functinal paraganglioma. *Heart* **2013**, *99*, 1438–1444.
4. Reister, A.; Weismann, D.; Quinkler, M.; Lichtenauer, U.D.; Sommerey, S.; Halbritter, R.; Penning, R.; Spitzweg, C.; Schopohl, J.; Beuschlein, F.; Reincke, M. Life-threatening events in patients with pheochromcytoma. *Eur. J. Endocrinol.* **2015**, *173*, 757–764.
5. Gu, Y.W.; Poste, J.; Kunal, M.; Schwarcz, M.; Weiss, I. Cardiovascular manifestations of pheochromocytoma. *Cardiol. Rev.* **2017**, *25*, 215–222.
6. Berends, A.M.A.; Kerstens, M.N.; Lenders, J.W.M.; Timmers, H.J.L.M. Approach to the Patient: Perioperative Management of the Patient with Pheochromocytoma or Sympathetic Paraganglioma. *J. Clin. Endocrinol. Metab.* **2020**, *105*, dgaa441.
7. Lenders, J.W.M.; Duh, Q.Y.; Eisenhofer, G.; Gimenez-Roqueplo, A.P.; Grebe, S.K.; Murad, M.H.; Naruse, M.; Pacak, K.; Young, W.F.; Endocrine Society. Pheochromocytoma and paraganglioma; an endocrine society clincal practice guideline. *J. Clin. Endocrinol. Metab.* **2014**, *99*, 1915–1942.
8. Buitenwerf, E.; Osinga, T.E.; Timmers, H.J.L.M.; Lenders, J.W.M.; Feelders, R.A.; Eekhoff, E.M.W.; Haak, H.R.; Corssmit, E.P.M.; Bisschop, P.H.L.T.; Valk, G.D.; et al. Efficacy of α-blockers on hemodynamic control during pheochromocytoma resection: A randomized controlled trial. *J. Clin. Endocrinol. Metab.* **2020**, *105*, 2381–2391.
9. Price, D.T.; Lefkowitz, R.J.; Caron, M.G.; Berkowitz, D.; Schwinn, D.A. Localization of mRNA for three distinct alpha 1-adrenergic receptor subtypes in human tissues: Implications for human alpha-adrenergic physiology. *Mol. Pharmacol.* **1994**, *45*, 171–175.
10. Rudner, X.L.; Berkowitz, D.E.; Booth, J.V.; Funk, B.L.; Cozart, K.L.; D'Amico, E.B.; El-Moalem, H.; Page, S.O.; Richardson, C.D.; Winters, B.; et al. Subtype specific regulation of human vascular alpha(1)-adrenergic receptors by vessel bed and age. *Circulation* **1999**, *100*, 2336–2343.
11. Guimarães, S.; Moura, D. Vascular adrenoceptors: An update. *Pharmacol. Rev.* **2001**, *53*, 319–356.
12. Flordellis, C.; Paris, H.; Karabinis, A.; Lymperopoulos, A. Pharmacogenomics of adrenoceptors. *Pharmacogenomics* **2004**, *5*, 803–817.
13. Docherty, J.R. Subtypes of functional alpha1-adrenoceptor. *Cell Mol. Life Sci.* **2010**, *67*, 405–417.
14. Giovannitti, J.A., Jr.; Thoms, S.M.; Crawford, J.J. Alpha-2 adrenergic receptor agonists: A review of current clinical applications. *Anesth. Prog.* **2015**, *62*, 31–39.
15. Docherty, J.R. Subtypes of functional alpha1- and alpha2-adrenoceptors. *Eur. J. Pharmacol.* **1998**, *361*, 1–15.
16. Flordellis, C.; Manolis, A.; Scheinin, M.; Paris, H. Clinical and pharmacological significance of alpha2-adrenoceptor polymorphisms in cardiovascular diseases. *Int. J. Cardiol.* **2004**, *97*, 367–372.
17. Small, K.M.; Liggett, S.B. Identification and functional characterization of alpha(2)-adrenoceptor polymorphisms. *Trends Pharmacol. Sci.* **2001**, *22*, 471–477.
18. Shastry, B.S. SNPs: Impact on gene function and phenotype. *Methods Mol. Biol.* **2009**, *578*, 3–22.
19. Lockette, W.; Ghosh, S.; Farrow, S.; MacKenzie, S.; Baker, S.; Miles, P.; Schork, A.; Cadaret, L. Alpha 2-adrenergic receptor gene polymorphism and hypertension in blacks. *Am. J. Hypertens.* **1995**, *8*, 390–394.
20. Svetkey, L.P.; Timmons, P.Z.; Emovon, O.; Anderson, N.B.; Preis, L.; Chen, Y.T. Association of hypertension with beta2- and alpha2c10-adrenergic receptor genotype. *Hypertension* **1996**, *27*, 1210–1215.
21. Freitas, S.R.; Pereira, A.C.; Floriano, M.S.; Mill, J.G.; Krieger, J.E. Association of alpha1a-adrenergic receptor polymorphism and blood pressure phenotypes in the Brazilian population. *BMC Cardiovasc. Disord.* **2008**, *8*, 40.
22. Sõber, S.; Org, E.; Kepp, K.; Juhanson, P.; Eyheramendy, S.; Gieger, C.; Lichtner, P.; Klopp, N.; Veldre, G.; Viigimaa, M.; et al. Targeting 160 candidate genes for blood pressure regulation with a genome-wide genotyping array. *PLoS ONE* **2009**, *4*, e6034.
23. Lei, B.; Morris, D.P.; Smith, M.P.; Svetkey, L.P.; Newman, M.F.; Rotter, J.I.; Buchanan, T.A.; Beckstrom-Sternberg, S.M.; Green, E.D.; Schwinn, D.A. Novel human alpha1a-adrenoceptor single nucleotide polymorphisms alter receptor pharmacology and biological function. *Naunyn Schmiedeberg's Arch Pharmacol.* **2005**, *371*, 229–239.
24. Buitenwerf, E.; Boekel, M.F.; van der Velde, M.I.; Voogd, M.F.; Kerstens, M.N.; Wietasch, G.J.K.G.; Scheeren, T.W.L. The hemodynamic instability score: Development and internal validation of a new rating method of intra-operative haemodynamic instability. *Eur. J. Anaesthesiol.* **2019**, *36*, 290–296.
25. Gabriel, S.B.; Schaffner, S.F.; Nguyen, H.; Moore, J.M.; Roy, J.; Blumenstiel, B.; Higgins, J.; DeFelice, M.; Lochner, A.; Faggart, M.; et al. The structure of haplotype blocks in the human genome. *Science* **2002**, *296*, 2225–2229.
26. Barrett, J.C.; Fry, B.; Maller, J.; Daly, M.J. Haploview: Analysis and visualization of LD and haplotype maps. *Bioinformatics* **2005**, *21*, 263–265.
27. Sinnwell, J.P.; Schaid, D.J. Haplo.Stats: Statistical Analysis of Haplotypes with Traits and Covariates when Linkage Phase is Ambiguous. R Package Version 1.8.6. 2020. Available online: https://CRAN.R-project.org/package=haplo.stats (accessed on 3 March 2021).

28. R Core Team R: A Language and Environment for Statistical Computing. R Foundation for Statistical Computing, Vienna, Austria, 2017. Available online: https://www.R-project.org (accessed on 3 March 2021).
29. Rosskopf, D.; Michel, M.C. Pharmacogenomics of G protein-coupled receptor ligands in cardiovascular medicine. *Pharmacol. Rev.* **2008**, *60*, 513–535.
30. Kurnik, D.; Muszkat, M.; Li, C.; Sofowora, G.G.; Friedman, E.A.; Scheinin, M.; Wood, A.J.; Stein, C.M. Genetic variations in the α(2A)-adrenoreceptor are associated with blood pressure response to the agonist dexmedetomidine. *Circ. Cardiovasc. Genet.* **2011**, *4*, 179–187.
31. Büscher, R.; Herrmann, V.; Ring, K.M.; Kailasam, M.T.; O'Connor, D.T.; Parmer, R.J.; Insel, P.A. Variability in phenylephrine response and essential hypertension: A search for human alpha(1B)-adrenergic receptor polymorphisms. *J. Pharmacol. Exp. Ther.* **1999**, *291*, 793–798.
32. Morrow, A.L.; Creese, I. Characterization of alpha 1-adrenergic receptor subtypes in rat brain: A reevaluation of [3H]WB4104 and [3H]prazosin binding. *Mol. Pharmacol.* **1986**, *29*, 321–330.
33. Adefurin, A.; Ghimire, L.V.; Kohli, U.; Muszkat, M.; Sofowora, G.G.; Li, C.; Levinson, R.T.; Paranjape, S.Y.; Stein, C.M.; Kurnik, D. Genetic variation in the $alpha_{1B}$-adrenergic receptor and vascular response. *Pharm. J.* **2017**, *17*, 366–371.
34. Kobilka, B.K.; Matsui, H.; Kobilka, T.S.; Yang-Feng, T.L.; Francke, U.; Caron, M.G.; Lefkowitz, R.J.; Regan, J.W. Cloning, sequencing, and expression of the gene coding for the human platelet alpha 2-adrenergic receptor. *Science* **1987**, *238*, 650–656.
35. Altman, J.D.; Trendelenburg, A.U.; MacMillan, L.; Bernstein, D.; Limbird, L.; Starke, K.; Kobilka, B.K.; Hein, L. Abnormal regulation of the sympathetic nervous system in alpha2A-adrenergic receptor knockout mice. *Mol. Pharmacol.* **1999**, *56*, 154–161.
36. Nunes, R.A.; Barroso, L.P.; Pereira Ada, C.; Krieger, J.E.; Mansur, A.J. Gender-related associations of genetic polymorphisms of α-adrenergic receptors, endothelial nitric oxide synthase and bradykinin B2 receptor with treadmill exercise test responses. *Open Heart* **2014**, *1*, e000132.
37. Small, K.M.; Brown, K.M.; Seman, C.A.; Theiss, C.T.; Liggett, S.B. Complex haplotypes derived from noncoding polymorphisms of the intronless alpha2A-adrenergic gene diversify receptor expression. *Proc. Natl. Acad. Sci. USA* **2006**, *103*, 5472–5477.
38. McCarthy, M.I.; Abecasis, G.R.; Cardon, L.R.; Goldstein, D.B.; Little, J.; Ioannidis, J.P.; Hirschhorn, J.N. Genome-wide association studies for complex traits: Consensus, uncertainty and challenges. *Nat. Rev. Genet.* **2008**, *9*, 356–369.
39. Burton, P.; Clayton, D.; Cardon, L.; Craddock, N.; Duncanson, A.; Kwiatkowski, D.; McCarthy, M.; Ouwehand, W.; Samani, N.; Todd, J.; et al. Association scan of 14,500 nonsynonymous SNPs in four diseases identifies autoimmunity variants. *Nat. Genet.* **2007**, *39*, 1329–1337.
40. Herlyn, P.; Müller-Hilke, B.; Wendt, M.; Hecker, M.; Mittlmeier, T.; Gradl, G.; Frequencies of polymorphisms in cytokines, neurotransmitters and adrenergic receptors in patients with complex regional pain syndrome type I after distal radial fracture. *Clin J Pain.* **2010**, *26*, 175–181.
41. Kelsey, R.M.; Alpert, B.S.; Dahmer, M.K.; Krushkal, J.; Quasney, M.W.; Alpha-adrenergic receptor gene polymorphisms and cardiovascular reactivity to stress in Black adolescents and young adults. *Psychophysiology* **2012**, *49*, 401–412.
42. Shorter, D.; Nielsen, D.A.; Huang, W.; Harding, M.J.; Hamon, S.C.; Kosten, T.R.; Pharmacogenetic randomized trial for cocaine abuse: disulfiram and α1A-adrenoceptor gene variation. *Eur. Neuropsychopharmacol.* **2013**, *23*, 1401–1407.
43. Wei, W.Q.; Feng, Q.; Weeke, P.; Bush W, Waitara, M.S.; Iwuchukwu, O.F.; Roden, D.M.; Wilke, R.A.; Stein, C.M.; Denny, J.C. Creation and Validation of an EMR-based Algorithm for Identifying Major Adverse Cardiac Events while on Statins. *AMIA Jt Summits Transl. Sci. Proc.* **2014**, *2014*, 112–119.
44. Adefurin, A.; Ghimire, L.V.; Kohli, U.; Muszkat, M.; Sofowora, G.G.; Li, C.; Paranjape, S.Y.; Stein, C.M.; Kurnik, D. Genetic variation in the α1A-adrenergic receptor and phenylephrine-mediated venoconstriction. *Pharm. J.* **2015**, *15*, 310–315.
45. Shorter D, Nielsen DA, Hamon SC, Nielsen EM, Kosten TR, Newton TF, De La Garza R 2nd. The α-1 adrenoceptor (ADRA1A) genotype moderates the magnitude of acute cocaine-induced subjective effects in cocaine-dependent individuals. *Pharm. Genom.* **2016**, *26*, 428–435.
46. Amorim Belo Nunes, R.; Pereira Barroso, L.; da Costa Pereira, A.; Pinto Brandão Rondon, M.U.; Negrão, C.E.; Krieger, J.E.; Mansur, A.J. Alpha2A-adrenergic receptor and eNOS genetic polymorphisms are associated with exercise muscle vasodilatation in apparently healthy individuals. *Int. J. Cardiol. Heart. Vasc.* **2016**, *13*, 14–18.
47. Han, J.; Zuo, J.; Zhu, D.; Gao, C. The correlation between SNPs within the gene of adrenergic receptor and neuropeptide Y and risk of cervical vertigo. *J. Clin. Lab. Anal.* **2018**, *32*, e22366.
48. Márquez, M.F.; Fragoso, J.M.; Pérez-Pérez, D.; Cázares-Campos, I.; Totomoch-Serra, A.; Gómez-Flores, J.R.; Vargas-Alarcón, G. Polymorphisms in β-adrenergic receptors are associated with increased risk to have a positive head-up tilt table test in patients with vasovagal syncope. *Rev. Invest. Clin.* **2019**, *71*, 124–132.
49. Elia, J.; Capasso, M.; Zaheer, Z.; Lantieri, F.; Ambrosini, P.; Berrettini, W.; Devoto, M.; Hakonarson, H. Candidate gene analysis in an on-going genome-wide association study of attention-deficit hyperactivity disorder: suggestive association signals in ADRA1A. *Psychiatr. Genet.* **2009**, *19*, 134–141.
50. Zhang, X.; Norton, J.; Carrière, I.; Ritchie, K.; Chaudieu, I.; Ryan, J.; Ancelin, M.L. Preliminary evidence for a role of the adrenergic nervous system in generalized anxiety disorder. *Sci. Rep.* **2017**, *7*, 42676.
51. Sun, Y.X.; Liao, Y.H.; Zhu, F.; Wang, M.; Chen, X.; Chen, F.; Cao, A.L.; Wang, J. Association between ADRA1A gene polymorphism and autoantibodies against the alpha1-adrenergic receptor in hypertensive patients. *Zhonghua Xin Xue Guan Bing Za Zhi* **2008**, *36*, 883–887. (In Chinese)

52. Mathias, R.A.; Grant, A.V.; Rafaels, N.; Hand, T.; Gao, L.; Vergara, C.; Tsai, Y.J.; Yang, M.; Campbell, M.; Foster, C.; et al.; A genome-wide association study on African-ancestry populations for asthma. *J. Allergy. Clin. Immunol.* **2010**, *125*, 336–346.e4.
53. Hawi, Z.; Matthews, N.; Barry, E. Kirley A, Wagner J, Wallace RH, Heussler HS, Vance A, Gill M, Bellgrove MA; l. A high density linkage disequilibrium mapping in 14 noradrenergic genes: evidence of association between SLC6A2, ADRA1B and ADHD. *Psychopharmacol.* **2013**, *225*, 895-902.
54. Orand, A.; Gupta, A.; Shih, W.; Presson, A.P.; Hammer, C.; Niesler, B.; Heendeniya, N.; Mayer, E.A.; Chang, L. Catecholaminergic Gene Polymorphisms Are Associated with GI Symptoms and Morphological Brain Changes in Irritable Bowel Syndrome. *PLoS One* **2015**, *10*, e0135910.
55. Parkman, H.P.; Mishra, A.; Jacobs, M.; Pathikonda M, Sachdeva P, Gaughan J, Krynetskiy E. . Clinical response and side effects of metoclopramide: associations with clinical, demographic, and pharmacogenetic parameters. *J. Clin. Gastroenterol.* **2012**, *46*, 494–503.
56. Shorter, D.I.; Zhang, X.; Domingo, C.B.; et al. Doxazosin treatment in cocaine use disorder: pharmacogenetic response based on an *alpha-1 adrenoreceptor subtype D* genetic variant. *Am. J. Drug Alcohol Abuse* **2020**, *46*, 184–193.
57. Lima, J.J.; Feng, H.; Duckworth, L.; Wang, J.; Sylvester, J.E.; Kissoon, N.; Garg, H. Association analyses of adrenergic receptor polymorphisms with obesity and metabolic alterations. *Metabolism* **2007**, *56*, 757–765.
58. Sickert, L.; Müller, D.J.; Tiwari, A.K.; Shaikh S, Zai C, De Souza R, De Luca V, Meltzer HY, Lieberman JA, Kennedy JL. Association of the alpha 2A adrenergic receptor -1291C/G polymorphism and antipsychotic-induced weight gain in European-Americans. Pharm. **2009**, *10*, 1169–1176.
59. de Cerqueira, C.C. ; Polina, E.R. ; Contini, V.; Marques, F.Z.; Grevet, E.H.; Salgado, C.A.; da Silva P.O.; Picon, F.A.; Belmonte-de-Abreu, P.; Bau, C.H. ADRA2A polymorphisms and ADHD in adults: possible mediating effect of personality. *Psychiatry Res.* **2011**, *186*, 345–350.
60. Yang, L.; Qian, Q.; Liu, L.; Li, H.; Faraone, S.V.; Wang, Y. Adrenergic neurotransmitter system transporter and receptor genes associated with atomoxetine response in attention-deficit hyperactivity disorder children. *J. Neural Transm.* **2013**, *120*, 1127–1133.
61. Lochman, J.; Balcar, V.J.; Sťastný, F.; Serý, O. Preliminary evidence for association between schizophrenia and polymorphisms in the regulatory Regions of the ADRA2A, DRD3 and SNAP-25 Genes. *Psychiatry Res.* **2013**, *205*, 7–12.
62. Rubin, D.H.; Althoff, R.R.; Ehli, E.A.; Davies, G.E.; Rettew, D.C.; Crehan, E.T.; Walkup, J.T.; Hudziak, J.J. Candidate gene associations with withdrawn behavior. *J. Child Psychol Psychiatr*y **2013**, *54*, 1337–1345.
63. McCracken, J.T.; Badashova, K.K.; Posey, D.J.; Aman, M.G.; Scahill, L.; Tierney, E.; Arnold, L.E.; Vitiello, B.; Whelan, F.; Chuang, S.Z.; et al. Positive effects of methylphenidate on hyperactivity are moderated by monoaminergic gene variants in children with autism spectrum disorders. *Pharm. J.* **2014**, *14*, 295–302.
64. Cummins, T.D.; Jacoby, O.; Hawi, Z.; Nandam, L.S.; Byrne, M.A.; Kim, B.N.; Wagner, J.; Chambers, C.D.; Bellgrove, M.A. Alpha-2A adrenergic receptor gene variants are associated with increased intra-individual variability in response time. *Mol. Psychiatry* **2014**, *19*, 1031–1036.
65. Kochetova, O.V.; Viktorova, T.V.; Mustafina, O.E.; Karpov, A.A.; Khusnutdinova EK. Genetic Association of ADRA2A and ADRB3 Genes with Metabolic Syndrome among the Tatars. *Genetika* **2015**, *51*, 830–834.
66. Kaabi, B.; Belaaloui, G.; Benbrahim, W.; Hamizi, K.; Sadelaoud, M.; Toumi, W.; Bounecer, H. ADRA2A Germline Gene Polymorphism is Associated to the Severity, but not to the Risk, of Breast Cancer. *Pathol. Oncol. Res.* **2016**, *22*, 357–365.
67. Gomez-Sanchez, C.I. ; Riveiro-Alvarez, R.; Soto-Insuga, V.; Rodrigo, M.; Tirado-Requero, P.; Mahillo-Fernandez, I.; Abad-Santos, F.; Carballo, J.J.; Dal-Ré, R.; Ayuso, C. Attention deficit hyperactivity disorder: genetic association study in a cohort of Spanish children. *Behav. Brain Funct.* **2016**, *12*, 2.
68. Hegvik, T.A.; Jacobsen, K.K.; Fredriksen, M.; Zayats, T.; Haavik, J. A candidate gene investigation of methylphenidate response in adult attention-deficit/hyperactivity disorder patients: results from a naturalistic study. *J. Neural Transm.* **2016**, *123*, 859–865.
69. Adefurin, A.; Darghosian, L.; Okafor, C.; Kawai. V.; Li, C.; Shah, A.; Wei, W.Q.; Kurnik, D.; Stein, C.M. Alpha2A adrenergic receptor genetic variation contributes to hyperglycemia after myocardial infarction. *Int. J. Cardiol.* **2016**, *215*, 482–486.
70. Havranek, M.M.; Hulka, L.M.; Tasiudi, E.; Eisenegger, C.; Vonmoos, M.; Preller, K.H.; Mössner, R.; Baumgartner, M.R.; Seifritz, E.; Grünblatt, E.; et al. α_{2A} -Adrenergic receptor polymorphisms and mRNA expression levels are associated with delay discounting in cocaine users. *Addict. Biol.* **2017**, *22*, 561–569.
71. Myer, N.M.; Boland, J.R.; Faraone, S.V. Pharmacogenetics predictors of methylphenidate efficacy in childhood ADHD. *Mol. Psychiatry* **2018**, *23*, 1929–1936.
72. Sokol, J.; Skerenova, M.; Ivankova, J.; Simurda, T.; Stasko, J. Association of Genetic Variability in Selected Genes in Patients With Deep Vein Thrombosis and Platelet Hyperaggregability. *Clin. Appl. Thromb. Hemost.* **2018**, *24*, 1027–1032.
73. Papathanasopoulos, A.; Camilleri, M.; Carlson, P.J.; Vella, A.; Nord, S.J.; Burton, D.D.; Odunsi, S.T.; Zinsmeister, A.R. A preliminary candidate genotype-intermediate phenotype study of satiation and gastric motor function in obesity. *Obesity* **2010**, *18*, 1201–1211.
74. Cho, S.C.; Kim, J.W.; Kim, H.W.; Kim, B.N.; Shin, M.S.; Cho, D.Y.; Jung, S.W.; Chung, U.S.; Son, J.W. Effect of ADRA2A and BDNF gene-gene interaction on the continuous performance test phenotype. *Psychiatr Genet.* **2011**, *21*,132–135.
75. Talmud, P.J.; Cooper, J.A.; Gaunt, T.; Holmes, M.V.; Shah, S.; Palmen, J.; Drenos, F.; Shah, T.; Kumari, M.; Kivimaki, M.; et al. Variants of ADRA2A are associated with fasting glucose, blood pressure, body mass index and type 2 diabetes risk: meta-analysis of four prospective studies. *Diabetologia* **2011**, *54*, 1710–1719.

76. Bo, S.; Cassader, M.; et al. The rs553668 polymorphism of the ADRA2A gene predicts the worsening of fasting glucose values in a cohort of subjects without diabetes. A population-based study. *Diabet. Med.* **2012**, *29*, 549–552.
77. Li T, Zhu X, Wu X, Li J, Pan L, Li P, Xin Z, Gu HF, Liu, Y. Evaluation of the association between the ADRA2A genetic polymorphisms and type 2 diabetes in a Chinese Han population. Genet Test Mol Biomarkers. 2012;16(12):1424-1427.
78. Mlakar V, Jurkovic Mlakar S, et al. ADRA2A is involved in neuro-endocrine regulation of bone resorption. J Cell Mol Med. 2015;19(7):1520-1529.
79. Nunes RAB, Lima LB, Tanaka NI, da Costa Pereira, A.; Krieger, J.E.; Mansur, A.J. Genetic associations of bradykinin type 2 receptor, alpha-adrenoceptors and endothelial nitric oxide synthase with blood pressure and left ventricular mass in outpatients without overt heart disease. Int J Cardiol Heart Vasc. 2018;21:45-49.
80. Xu, D.; Liu, L.; Li, H.; Sun, L.; Yang, L.; Qian, Q.; Wang, Y. Potential Role of *ADRA2A* Genetic Variants in the Etiology of ADHD Comorbid With Tic Disorders. *J. Atten. Disord.* **2021**, *25*, 33–43.
81. Leońska-Duniec, A.; Jastrzębski, Z.; Jażdżewska, A.; et al. Individual Responsiveness to Exercise-Induced Fat Loss and Improvement of Metabolic Profile in Young Women is Associated with Polymorphisms of Adrenergic Receptor Genes. *J. Sports Sci. Med.* **2018**, *17*,134–144.
82. Totomoch-Serra. A. Perez-Muñoz, A.; Diaz-Badillo, Á. The ADRA2A rs553668 variant is associated with type 2 diabetes and five variants were associated at nominal significance levels in a population-based case-control study from Mexico City. *Gene* **2018**, *669*, 28–34.
83. Mărginean, C.O.; Mărginean, C.; Bănescu, C.; Meliţ, L.E.; Tripon, F.; Iancu, M. The relationship between MMP9 and ADRA2A gene polymorphisms and mothers-newborns' nutritional status: an exploratory path model (STROBE compliant article). *Pediatr. Res.* **2019**, *85*, 822–829.
84. Perroud, N.; Aitchison, K.J.; Uher, R.; Smith, R.; Huezo-Diaz, P.; Marusic, A.; Maier, W.; Mors, O.; Placentino, A.; Henigsberg, N. et al. Genetic predictors of increase in suicidal ideation during antidepressant treatment in the GENDEP project. Neuropsychopharmacol. **2009**, *34*, 2517–2528.
85. Song, Y.; Tang, X.F.; Yao, Y.; He, C.; Xu, J.J.; Wang, H.H.; Gao, Z.; Wang, M.; Yuan, J.Q. Association of α2A-Adrenergic Receptor Genetic Variants with Platelet Reactivity in Chinese Patients on Dual Antiplatelet Therapy Undergoing Percutaneous Coronary Intervention. *Biomed. Environ. Sci.* **2017**, *30*, 898–906.
86. Clarke, T.K.; Dempster, E.; Docherty, S.J. Desrivieres S, Lourdsamy A, Wodarz N, Ridinger M, Maier W, Rietschel M, Schumann G. Multiple polymorphisms in genes of the adrenergic stress system confer vulnerability to alcohol abuse. *Addict. Biol.* **2012**, *17*, 202–208.
87. Hiltunen, T.P.; Donner. K.M.; Sarin, A.P.; Saarela, J.; Ripatti, S.; Chapman, A.B.; Gums, J.G.; Gong, Y.; Cooper-DeHoff, R.M.; Frau, F.; et al. Pharmacogenomics of hypertension: a genome-wide, placebo-controlled cross-over study, using four classes of antihypertensive drugs. *J. Am. Heart Assoc.* **2015**, *4*, e001521.
88. Muszkat, M.; Kurnik, D.; Sofowora, G.G. Solus J, Xie, H.G.; Harris, P.A.; Williams, S.M.; Wood, A.J.; Stein, C.M. Desensitization of vascular response in vivo: contribution of genetic variation in the [alpha]2B-adrenergic receptor subtype. *J. Hypertens.* **2010**, *28*, 278–284.
89. De Fusco, M.; Vago, R.; Striano, P.; Di Bonaventura, C.; Zara, F.; Mei, D.; Kim, M.S.; Muallem, S.; Chen, Y.; Wang, Q.; et al. The α2B-adrenergic receptor is mutant in cortical myoclonus and epilepsy. *Ann. Neurol.* **2014**, *75*, 77–87.

Supplementary Table S2: **Standardized incremental dosage steps for doxasozin and phenoxybenzamine**

**Clinical step**	**Classification dosage step**	**Doxasozin (mg)**	**Phenoxybenzamine (mg)**
1	Low	4	10
2	Low	8	20
3	Moderate	12	40
4	Moderate	16	60
4.5	Moderate	20	70
5	Moderate	24	80
5.5	Moderate	28	90
6	High	32	100
6.5	High	36	110
7	High	40	120
8	High	48	140

Supplementary Table S3: Sensitivity analyses for dose of α-adrenergic receptor blockers

**SNP-allele**	**All samples (n=116)**		**Call rate > 50% (n=110)**		**Europeans (n=107)**	
	**OR**	**p-value**	**OR**	**p-value**	**OR**	**p-value**
rs2229169-T	0.73	0.39	0.70	0.33	0.69	0.34
rs2030373-C	0.52	0.17	0.52	0.17	0.68	0.43
rs6884105-G	0.67	0.31	0.67	0.31	0.83	0.65
rs756275-T	0.70	0.59	0.71	0.62	0.56	0.40
rs6892282-T	1.35	0.39	1.35	0.39	1.09	0.81
rs10515807-G	0.31	0.047*	0.31	0.045*	0.37	0.089
rs6888306-T	0.89	0.77	0.91	0.80	0.66	0.33
rs13162302-G	1.03	0.94	1.04	0.92	0.90	0.80
rs11750092-T	1.20	0.66	1.21	0.63	1.07	0.88
rs3802241-G	1.28	0.48	1.19	0.63	1.49	0.31
rs1048101-T	1.43	0.26	1.48	0.27	1.73	0.10
rs13278849-G	0.92	0.80	0.99	0.98	0.95	0.89
rs17426222-T	2.04	0.12	1.95	0.14	1.79	0.22
rs4732957-C	1.50	0.34	1.42	0.41	1.42	0.41

rs4732682-T	0.78	0.50	0.75	0.44	0.90	0.79
rs573514-G	1.92	0.086	1.97	0.076	1.76	0.15
rs1383914-T	1.41	0.29	1.46	0.25	1.21	0.58
rs3808585-T	0.74	0.46	0.77	0.53	0.64	0.28
rs521674-T	3.30	0.014*	3.30	0.014*	2.81	0.040*
rs1800544-G	2.01	0.072	2.13	0.056	1.79	0.15
rs1800545-A	1.34	0.61	1.55	0.44	1.08	0.90
rs553668-G	0.26	0.024*	0.24	0.019*	0.32	0.061
rs2236554-T	0.85	0.69	0.85	0.69	0.65	0.31
rs1556832-T	0.63	0.20	0.67	0.28	0.55	0.12

OR, odds ratio, *, nominal significant

Supplementary Table S4: Sensitivity analyses for number of side effects

SNP-allele	All samples (n=116)		Call rate > 50% (n=110)		Europeans (n=107)	
	OR	p-value	OR	p-value	OR	p-value
rs2229169-T	0.98	0.95	0.97	0.91	0.98	0.93
rs2030373-C	0.58	0.14	0.58	0.14	0.76	0.48
rs6884105-G	0.95	0.85	0.95	0.85	1.10	0.74
rs756275-T	1.01	0.98	0.96	0.94	1.10	0.87
rs6892282-T	1.58	0.10	1.57	0.10	1.36	0.28
rs10515807-G	0.27	0.005*	0.27	0.006*	0.26	0.006*
rs6888306-T	1.23	0.52	1.20	0.56	0.99	0.97
rs13162302-G	1.27	0.47	1.24	0.51	1.07	0.84
rs11750092-T	1.36	0.36	1.33	0.40	1.15	0.70
rs3802241-G	1.19	0.51	1.16	0.57	1.14	0.63
rs1048101-T	0.99	0.96	1.03	0.91	0.99	0.98
rs13278849-G	1.12	0.68	1.12	0.70	1.18	0.57
rs17426222-T	1.16	0.64	1.13	0.71	1.13	0.71
rs4732957-C	1.06	0.87	1.09	0.81	0.93	0.84
rs4732682-T	0.95	0.86	0.96	0.87	0.95	0.85
rs573514-G	1.04	0.90	1.02	0.94	1.11	0.72
rs1383914-T	1.21	0.43	1.15	0.57	1.23	0.44
rs3808585-T	1.31	0.36	1.28	0.40	1.24	0.47
rs521674-T	1.04	0.91	1.04	0.91	1.17	0.65
rs1800544-G	1.23	0.48	1.07	0.83	1.35	0.33
rs1800545-A	0.87	0.75	0.81	0.62	0.91	0.84
rs553668-G	0.72	0.39	0.74	0.43	0.66	0.30
rs2236554-T	1.11	0.73	1.11	0.73	0.98	0.96
rs1556832-T	0.87	0.58	0.82	0.45	0.89	0.67

OR, odds ratio, *, nominal significant.
